# Global Invasive Bacterial Vaccine-Preventable Diseases Surveillance — 2008–2014

**Published:** 2014-12-12

**Authors:** Jillian Murray, Mary Agócs, Fatima Serhan, Simarjit Singh, Maria Deloria-Knoll, Katherine O’Brien, Jason M. Mwenda, Richard Mihigo, Lucia Oliveira, Nadia Teleb, Hinda Ahmed, Annemarie Wasley, Dovile Videbaek, Pushpa Wijesinghe, Arun Bhadra Thapa, Kimberly Fox, Fem Julia Paladin, Rana Hajjeh, Stephanie Schwartz, Chris Van Beneden, Terri Hyde, Claire Broome, Thomas Cherian

**Affiliations:** 1 Department of Immunization, Vaccines, and Biologicals, World Health Organization (WHO), Geneva, Switzerland; 2 IVAC, Johns Hopkins University; 3 WHO Regional Office for Africa, Brazzaville, Republic of the Congo; 4 WHO Regional Office for the Americas, District of Columbia, United States; 5 WHO Regional Office for the Eastern Mediterranean, Cairo, Egypt; 6 WHO Regional Office for Europe, Copenhagen, Denmark; 7 WHO Regional Office for South-East Asia, New Delhi, India; 8 WHO Regional Office for the Western Pacific, Manila, Philippines; 9 Division of Bacterial Diseases, National Center for Immunization and Respiratory Diseases, CDC; 10 Global Immunization Division, Center for Global Health, CDC; 11 Emory University, Atlanta, Georgia

Meningitis and pneumonia are leading causes of morbidity and mortality in children globally infected with *Streptococcus pneumoniae* (pneumococcus), *Neisseria meningitidis,* and *Haemophilus influenzae* causing a large proportion of disease. Vaccines are available to prevent many of the common types of these infections. *S. pneumoniae* was estimated to have caused 11% of deaths in children aged <5 years globally in the pre-pneumococcal conjugate vaccine (PCV) era (1). Since 2007, the World Health Organization (WHO) has recommended inclusion of PCV in childhood immunization programs worldwide, especially in countries with high child mortality (2). As of November 26, 2014, a total of 112 (58%) of all 194 WHO member states and 44 (58%) of the 76 member states ever eligible for support from Gavi, the Vaccine Alliance (Gavi), have introduced PCV. Invasive pneumococcal disease (IPD) surveillance that includes data on serotypes, along with meningitis and pneumonia syndromic surveillance, provides important data to guide decisions to introduce PCV and monitor its impact.

## Sentinel Hospital Surveillance Network for Invasive Bacterial Vaccine-Preventable Diseases

In 2008, WHO brought together 91 sentinel hospital sites in existing regional surveillance networks in 36 WHO member states to strengthen, standardize, and expand a global network conducting sentinel hospital surveillance for invasive bacterial vaccine-preventable diseases (IB-VPD). The objectives of the network were to 1) collect data to describe the epidemiology and estimate the burden of IB-VPD, 2) establish a surveillance platform to measure impact after introduction of *Haemophilus influenzae* type b vaccine or PCV, and 3) detect and characterize the circulating bacterial types.

The global IB-VPD surveillance network includes sentinel hospitals and laboratories that report clinical and laboratory data on cases of suspected meningitis,* pneumonia,† or sepsis§ among children aged <5 years to the national ministries of health and WHO. All sites conduct surveillance for meningitis (Tier 1); some sites also investigate cases of pneumonia and sepsis (Tier 2), and a few conduct population-based surveillance for all three diseases, permitting incidence estimates (Tier 3). At all hospitals, cerebrospinal fluid is collected from suspected meningitis patients per routine clinical practice and tested at the site by Gram stain, culture, and, where available, a rapid diagnostic test (immunochromatographic or latex agglutination). Blood cultures are performed in suspected pneumonia and sepsis cases. Cerebrospinal fluid specimens and isolates are sent to reference laboratories for polymerase chain reaction testing, confirmation, and serotyping.

During 2008–2012, WHO and partners implemented a comprehensive plan to enhance the network’s capacity to collect and analyze data, including development of protocols for standardizing surveillance and collaborations with regional and global reference laboratories. WHO worked with ministries of health to coordinate the provision of technical assistance and laboratory supplies to sentinel hospitals with help from various partner organizations. CDC and Johns Hopkins University provided technical assistance in development of protocols and analysis. WHO also provided financial support to Gavi-eligible countries and coordinated an annual external quality assessment program for participating laboratories, consisting of the distribution of external quality assessment panels and confirmatory testing of a subset of samples exchanged between regional and global reference laboratories. Data collected from network participants are shared biannually via global surveillance feedback bulletins (3). To provide guidance for improvement and standardization of the global network, WHO established both an informal technical advisory group of experts for new vaccines surveillance and a laboratory technical working group.

## Sentinel Network Review by Technical Advisory Group, 2013

In 2013, WHO, the informal technical advisory group, and partners undertook a strategic review to assess network performance and inform future activities. The review cited progress made while highlighting challenges of conducting IB-VPD surveillance such as low bacterial isolation rates. The network met several of the original objectives: countries established Tier 1 surveillance in all WHO regions, Tier 2 in four, and Tier 3 in two regions (Table 1). The network served as a platform for special studies now being implemented in several countries (e.g., PCV impact on pneumonia diagnosed by chest radiography in Mongolia). Data also were used to support evidence-based decision-making for introduction of PCVs into national immunization programs in several countries.

By 2012, the network had expanded to 150 sites in 58 countries; however, the quality and consistency of the resulting data varied markedly by sentinel site. The review noted that significant changes were necessary to produce data of adequate quality to document vaccine impact, including a more focused approach in both the size and key objectives of the network. The WHO Strategic Advisory Group of Experts on Immunization recommended prioritizing the monitoring of PCV impact and targeting resources to support a smaller number of higher-performing sites, emphasizing quality of surveillance sufficient to monitor PCV impact on disease (4,5). Given the challenges of etiologic diagnosis, the WHO Strategic Advisory Group of Experts on Immunization also suggested that additional approaches to ensuring availability of national data for decision-making should be explored, such as data on in-patient pediatric pneumonia.

## Network Status, 2014

In response to the WHO Strategic Advisory Group of Experts on Immunization recommendation, the performance of each of the 150 sites reporting data to WHO in 2012 was evaluated to identify higher-performing sites using the following criteria: 1) reporting data to WHO for ≥10 months annually and 2) enrolling ≥100 suspected meningitis cases (Tier 1) or ≥500 suspected meningitis, pneumonia or sepsis cases (Tier 2) annually for ≥2 years during 2010–2012. Based on this evaluation, 56 of the sites in Gavi-eligible countries were selected for targeted technical and financial support. In addition, 10 new or higher performing Gavi-eligible sites were included (Figure, Table 1). Sites not receiving targeted support were encouraged to continue reporting data to WHO as part of the IB-VPD network.

As of July 2014, 130 sites in 57 countries reported 2013 data to WHO, including 63 sites in 38 Gavi-eligible countries selected for targeted support in 2014 and 2015 (Figure, Table 1). Among 38 countries with a site receiving targeted support and reporting 2013 data, nine (24%) have not yet introduced PCV. During 2009–2013, 94,871 hospitalized children were enrolled in surveillance in targeted sites (Table 2). During 2013, a total of 574 children had one of the three potentially vaccine-preventable pathogens detected. Among 511 children with meningitis, 69% were infected with *S. pneumoniae*, 17% *H. influenzae*, and 14% *N. meningitidis*; among 63 children with pneumonia or sepsis, 83% had *S. pneumoniae* and 17% *H. influenzae*.

Areas of ongoing work to improve IB-VPD surveillance include 1) uniformly instituting “zero reporting” to differentiate zero cases detected from lack of reporting, 2) moving all sites from aggregate to case-based reporting, 3) focusing on improved quality assurance in laboratory testing and reporting, 4) piloting a web-based data management system, 5) improving laboratory methods, and 6) collecting serotype/serogroup data to determine what proportions of *S. pneumoniae*, *N. meningitidis*, and *H. influenzae* detected by surveillance are vaccine-preventable. In addition, routine use of unique case identification numbers is being implemented to improve linkage of off-site laboratory data with clinical data.

### Discussion

IPD surveillance has provided scientific data needed to advocate for PCV introduction in some countries and will continue to be useful in supporting decision-making in countries that have not yet introduced PCV. The IB-VPD surveillance network has made progress in advancing IPD surveillance but has encountered many challenges. Consistent case reporting and accurate implementation of bacterial diagnostics at hospitals in resource-limited areas, especially culture and isolation of organisms, remains difficult. Further analysis of network data is under way to determine site capacity to identify probable bacterial meningitis cases¶ and to assess the additional IPD cases identified by polymerase chain reaction testing at reference laboratories.

Surveillance must be consistent for a minimal time period; ideally at least 2 years of data prevaccine and 5 years of data postvaccine introduction are recommended to accurately assess vaccine impact. Many network sites that have not yet introduced PCV have an opportunity to strengthen baseline surveillance capacity and quality. These sites can document the presence of pneumococcus to build evidence for PCV introduction and to establish a baseline for measuring PCV impact on meningitis and pneumonia syndromes, IPD, and serotype distribution. Limited resources have been focused on carefully selected sentinel hospital sites to increase the chances of success. Most network countries will not be able to assess serotype-replacement, which requires data on the IPD incidence caused by vaccine and nonvaccine serotypes.

Despite the absence of quality baseline surveillance data in some countries that have already introduced PCV, vaccine impact might be estimated using other study designs. Areas with limited laboratory capacity might be able to document impact against the principal clinical syndromes caused by pneumococcus. Sites with consistent pneumonia case enrollment and well-characterized clinical data are assessing the feasibility of special studies to document the impact of PCV on pneumonia incidence. Investigators also might explore PCV impact monitoring by analysis of administrative data tracking hospitalizations for pediatric pneumonia.

The capacity established by the surveillance network to systematically enroll cases, collect clinical information, conduct microbiologic investigation, and analyze data has value beyond the immediate objective of documenting the impact of current vaccines. If the capacity is enhanced, it might facilitate the conduct of other studies and provide a platform at these sentinel hospitals to establish surveillance for other vaccine preventable diseases such as typhoid fever and congenital rubella syndrome.

What is already known on this topic?

Meningitis and pneumonia are leading causes of morbidity and mortality in children globally. Since 2007, the World Health Organization (WHO) has recommended inclusion of pneumococcal conjugate vaccine (PCV) in childhood immunization programs worldwide, especially in countries with high child mortality.

What is added by this report?

The WHO invasive bacterial and vaccine-preventable disease (IB-VPD) surveillance network includes sentinel hospitals and laboratories that report clinical and laboratory data on cases of suspected meningitis, pneumonia, or sepsis among children aged <5 years to national ministries of health and WHO. As of November 26, 2014, 112 (58%) of all 194 WHO member states and 44 (58%) of the 76 member states ever eligible for support from Gavi have introduced PCV.

What are the implications for public health practice?

IB-VPD sentinel hospital surveillance that includes case-based data with laboratory confirmation information, along with meningitis and pneumonia syndromic surveillance, provides important data to guide decisions to introduce PCV and monitor its impact. The strategic review of the WHO IB-VPD network determined that this program is useful for country decision-making around vaccine usage. As more countries introduce PCV, it is important for this network to continue to improve to be able to assess the impact of this vaccine globally and act as a platform for surveillance of other diseases.

## Figures and Tables

**FIGURE:**
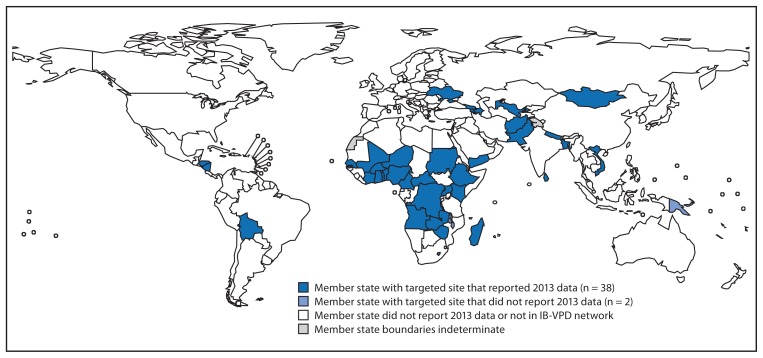
World Health Organization member states with at least one invasive bacterial vaccine-preventable diseases (IB-VPD) hospital sentinel surveillance site receiving targeted support — 2014 and 2015^*^ ^*^ Data reported at July 2014.

**TABLE 1:** Characteristics of global invasive bacterial vaccine-preventable diseases (IB-VPD) sentinel surveillance network sites that reported data to the World Health Organization (WHO), by WHO region — 2013*

WHO region	All sites reporting 2013 data				Sites targeted for support during 2014 and 2015†							
	
	Sentinel sites		Member states with a reporting sentinel site		Sentinel sites		Site reported 2013 data					

							Sentinel sites	Member states	Member state introduced PCV	Type of surveillance§		
						
	No.	%	No.	%	No.	%	No.	No.	No.	Tier 1	Tier 2	Tier 3
Africa	45	35	29	51	33	50	32	21	19	32	0	0
Americas	29	22	10	18	5	8	5	3	3	0	5	0
Eastern Mediterranean	23	18	6	11	11	17	11	4	4	8	3	0
Europe	14	11	6	11	7	11	7	5	3	7	0	0
South-East Asia	5	4	3	5	6	9	5	3	0	0	4	1
Western Pacific	14	11	3	5	4	6	3	2	0	2	0	1¶
**Total**	**130**	**100**	**57**	**100**	**66**	**100**	**63**	**38**	**29**	**49**	**12**	**2**

**Abbreviation:** PCV = pneumococcal conjugate vaccine.

* Data reported at July 2014.

† Higher performing sites located in Gavi-eligible WHO member states targeted to receive technical and financial support.

§ Tier 1: sites conduct surveillance for meningitis cases only (cerebrospinal fluid [CSF] collected). Tier 2: sites conduct surveillance for meningitis, pneumonia, and sepsis cases (CSF and blood collected). Tier 3: sites conduct population-based surveillance for meningitis, pneumonia and sepsis (CSF and blood collected).

¶ Six hospitals in Mongolia comprise one Tier 3 surveillance site.

**TABLE 2:** Characteristics of children aged <5 years who were admitted to sentinel hospitals receiving targeted support in the World Health Organization (WHO) global invasive bacterial vaccine-preventable diseases network, by WHO region — 2009–2013*

WHO region	Tier 1 meningitis surveillance		Tiers 2 and 3† pneumonia and sepsis surveillance	
	
	No. of children with suspected meningitis who had cerebrospinal fluid collected	Range by site	No. of children with suspected pneumonia and sepsis who had blood collected	Range by site
Africa	31,091	177–4,276	N/A	N/A
Americas	566	1–76	4,839	68–1,027
Eastern Mediterranean	15,058	192–4,038	2,297	151–997
Europe	1,065	16–394	N/A	N/A
South-East Asia	7,064	11–2,705	17,886	183–6,507
Western Pacific	1,794	5–882	13,211	34–3,525
**Total**	**56,638**	**5–4,485**	**38,233**	**34–6,507**

**Abbreviation:** N/A = not available.

* Data reported as of July 2014.

† Meningitis cases enrolled at Tier 2 and 3 sites are included in the Tier 1 case counts.

## Footnotes

* The suspected meningitis case definition is either 1) the sudden onset of fever (>101.3°F [38.5°C] rectal or 100.4°F [38.0°C] axillary) and one of the following signs: neck stiffness, altered consciousness with no other alternative diagnosis, or other meningeal sign in a child aged 0–59 months or 2) a clinical diagnosis of meningitis in a hospitalized child aged aged 0–59 months.

† The suspected pneumonia case definition is coughing or difficulty breathing and tachypnea when calm at a rate of ≥60 breaths/min in an infant aged <2 months, ≥50 breaths/min in an infant aged 2 to <12 months, or ≥40 breaths/min in a child aged 12–59 months.

§ The suspected sepsis case definition is presence of at least two of the following danger signs and without meningitis or pneumonia clinical syndrome in a child aged 12–59 months: inability to drink or breastfeed, vomiting everything, convulsions (except in malaria endemic areas), prostration/lethargy (abnormally sleepy or difficult to wake), severe malnutrition, and hypothermia (≤96.8°F [36.0°C]).

¶ An enrolled suspected meningitis patient with cerebral spinal fluid examination showing turbid appearance or leukocytosis (>100 cells/*μ*L) or both leukocytosis (10–100 cells/*μ*L) and either an elevated protein (>100 mg/dL) or decreased glucose (<40 mg/dL). If protein and glucose results are not available, diagnosis is based on turbid appearance or leukocytosis >100 cells/*μ*L).

## References

[1] O’Brien K, Wolfson LJ, Watt JP (2009). Burden of disease caused by *Streptococcus pneumoniae* in children younger than 5 years: global estimates. Lancet.

[2] World Health Organization (2007). Pneumococcal conjugate vaccine for childhood immunization—WHO position paper. Wkly Epidemiol Rec.

[3] World Health Organization (2014). NUVI — resources for monitoring and surveillance.

[4] World Health Organization (2014). Meeting of the Strategic Advisory Group of Experts on Immunization, November 2013–conclusions and recommendations. Wkly Epidemiol Rec.

[5] World Health Organization (2014). Strategic Advisory Group of Experts (SAGE) on Immunization.

